# Younger patients and smokers report a higher level of pain after knee arthroscopy: a clinical and experimental study including synovial metabolism

**DOI:** 10.1007/s00167-018-5125-x

**Published:** 2018-09-07

**Authors:** Lukas Berglund, Anders Stålman, Elisabeth Dungner, Abdul Rashid Qureshi, Maritha Kumlin, Li Felländer-Tsai

**Affiliations:** 1 Division of Orthopaedics and Biotechnology, Department of Clinical Science Intervention and Technology (CLINTEC), Karolinska Institutet and Karolinska University Hospital, Stockholm, Sweden; 2 Division of Renal Medicine, Karolinska Institutet and Karolinska University Hospital, Stockholm, Sweden; 3 Capio Artro Clinic, Stockholm Sports Trauma Research Centre, Sophiahemmet, Karolinska Institutet, Stockholm, Sweden

**Keywords:** Pain, Postoperative, Arthroscopy, Microdialysis, Synovial membrane, Forecasting, Visual analogue scale, Prostaglandins E, Glycerol, Glucose

## Abstract

**Purpose:**

Factors associated with post-surgical pain are not fully explored. The aim of this study was to identify determinants of postoperative pain after arthroscopic surgery of the knee. Synovial tissue metabolism was analysed by microdialysis and the association with individual and peri-surgical factors to identify determinants important for pain management and thus patient satisfaction.

**Methods:**

Cross-sectional study of 57 patients (22 women) with median age of 39 years. All patients were operated on with arthroscopic surgery of the knee and monitored postoperatively with synovial microdialysis. The cross-sectional cohort was investigated to determine local tissue levels of inflammatory and metabolic compounds along with postoperative pain experience. Measurements: pain was determined by visual analogue scale (VAS). Postoperative synovial tissue levels of prostaglandin E_2_ (PGE_2_), glucose, and glycerol were measured by microdialysis in the knee synovium. Patients reporting VAS ≥ 4 received rescue pain medication with systemic opioids.

**Results:**

Initial results indicated that patients with pain (interpreted as having VAS ≥ 4), i.e. those receiving rescue medication with systemic opioids, were of a younger age (*p* = 0.04), lower body weight (*p* = 0.02), had a lower BMI (*p* = 0.04) and/or were smokers (*p* = 0.02). A closer analysis using multinomial logistic regression showed a significantly higher amount of pain in smokers (*p* = 0.01) and patients of a younger age (*p* = 0.02). A significant correlation was also found between VAS and duration of surgery (*p* = 0.007). No significant correlation could be found between VAS and synovial levels of PGE_2_, glycerol and glucose, but a statistically significant decline with time of PGE_2_ in both groups.

**Conclusions:**

The results from this study show a significantly higher frequency of pain, post-surgery among younger patients (*p* = 0.02) and smokers (*p* = 0.01), as well as an association between pain and length of surgery (*p* = 0.007). These findings point out individual factors useful for the prediction of postoperative pain after arthroscopic surgery and are clinically important for personalized pain management.

**Level of evidence:**

II.

## Introduction

In modern healthcare, the number of surgeries done at outpatient clinics has significantly increased [1]. In the US, 51% of the total number of arthroscopic surgeries were made in freestanding ambulatory surgery centres, with an overall increase of 49% between 1996 and 2006 [1]. With this comes a need for both patients and surgeons to optimize pain management to promote early discharge and optimal mobilization. The mechanisms for postoperative pain are complex, multifactorial, and subjected to the individual patients’ perioperative experience [2]. Ways of predicting pain, and patients prone to pain, have thus far been difficult. Social factors and status are known to affect frequency and level of pain [3–7], but attempts to correlate postoperative pain to factors such as preoperative stress, etc. have not been successful [8]. Metabolites such as prostaglandin E_2_, glycerol and glucose have in earlier studies been measured and shown to correlate to changes in the synovial membrane of the knee making it possible to evaluate local metabolic states [9–13]. The metabolites have to our knowledge not yet been a part of forecasting postoperative pain. The present study hypothesized that conditions pre- and during surgery including synovial metabolites may predict the degree of pain post-arthroscopic surgery. The aim of the study was to identify individual factors and localized metabolites as determinants of postoperative pain to improve and personalize future pain management.

## Materials and methods

The study design was observational cross-sectional cohort with surgery performed on eligible patients when operating room and laboratory personnel were available for performing microdialysis. The study period was between 2004 and 2013. The protocols for perioperative treatment, anaesthesia and the surgical procedures remained the same during the study. The study was conducted at the Department of Orthopaedic Surgery at Karolinska University Hospital in Huddinge, Sweden, with a catchment area representative of the general population.

### Patients

57 patients (22 women), healthy, apart from knee problems, were eligible for inclusion in the study. Informed written consent was obtained from each patient before inclusion.

The inclusion criteria were indication for arthroscopic surgery of the knee due to intra-articular pathology (ACL reconstruction, meniscal repair/resection or diagnostic surgery) using the International Statistical Classification of Diseases and Related Health Problems (ICD-10). Patients with the following diagnoses were included in the study. patients undergoing arthroscopic surgery: derangement of meniscus due to old tear or injury (23), tear of meniscus, current injury (5), unilateral primary osteoarthritis of knee (2), other unilateral secondary osteoarthritis of knee (1), tear of articular cartilage of knee, current (3), damage to multiple structures of the knee joint (2), sprain of the superior tibiofibular joint and ligament (2), late-onset pain and discomfort after injury to the lower extremity (1), late-onset pain and discomfort after injury to tendon or muscle in the lower extremity (1), and sprain of cruciate ligament of knee (1).

Performed type of arthroscopic surgery: arthroscopic partial excision of a meniscus in the knee joint (26), Arthroscopic partial excision of cartilage in the knee joint (2), arthroscopic synovectomy in the knee joint (1), arthroscopic exploration of the knee joint (8), arthroscopic or endoscopic reimplantation of a meniscus in the knee joint (3), other arthroscopic or endoscopic surgery on synovia or articulating surface (1).

Diagnoses of patients with a cruciate ligament injury: sprain of cruciate ligament of knee (15) and damage to multiple structures of the knee joint (1). Performed surgery: arthroscopic reconstruction of a ligament in the knee joint without using a foreign material (16). ACL reconstructive surgery was done using the ipsilateral semitendinosus tendon as autograft. Exclusion criteria were known systemic inflammatory/metabolic diseases, or a regular intake of anti-inflammatory or other drugs known to or suspected of altering the metabolism of the knee joint. Patient characteristics are shown in Table 1.

**Table 1:** Baseline characteristics of the study population

Variables	Rescue medication with opioids (RM) (*n* = 10)	No rescue medication with opioids (NRM) (*n* = 47)	*p* value
Age (years)	29 (18–40)	41 (18–58)	0.04*
Female, *n* (%)	5 (50)	17 (36)	n.s
Height (cm)	174 (165–190)	178 (162–188)	n.s
Weight (kg)	71 (64–87)	82 (64–106)	0.02*
Body mass index (kg/m^2^)	24 (22–29)	26 (21–34)	0.04*
Operating time (min)	39 (16–108)	34 (20–138)	n.s
ACL/arthroscopy, *n* (%)	2/8 (20)	14/33 (30)	n.s
Tourniquet, yes/no, *n* (%)	2/8 (20)	18/29 (38)	n.s
Cryo cuff, yes/no, *n* (%)	2/8 (20)	14/33 (42)	n.s
Osteoarthritis, yes/no, *n* (%)	2/8 (20)	17/30 (36)	n.s
Smoking, yes/no, *n* (%)	5/5 (50)	7/40 (15)	0.02*

Continuous variables are presented as median (10–90 percentiles). Categorical variables are presented as percentage

Statistical significance (*) was set at the level of *p* < 0.05. Patients with rescue medication were significantly younger (*p* = 0.04), with a lower body weight (*p* = 0.02), a lower BMI (*p* = 0.04) and a higher frequency of smoking (*p* = 0.02)

### Procedure

All patients received a standardized premedication of paracetamol (2000 mg) and codeine (60 mg). The patients were not allowed glucose infusion, NSAID, or cortisone perioperatively. Anaesthesia was introduced and maintained using atropine 0.5 mg/ml, propofol 10 mg/ml, fentanyl 0.05 mg/ml and sevoflurane. The duration of the knee arthroscopic surgical procedure was 13–160 min.

### Microdialysis

The method works by inserting a catheter with a tubular semipermeable membrane at its tip. When inserted, the catheter mimics the function of a capillary vessel by causing an equilibration across the semipermeable membrane between the interstitial fluid and dialysis fluid streaming through the probe. The equilibrated dialysis fluid is thereafter collected in a vial, and later analysed.

**Fig. 1:**
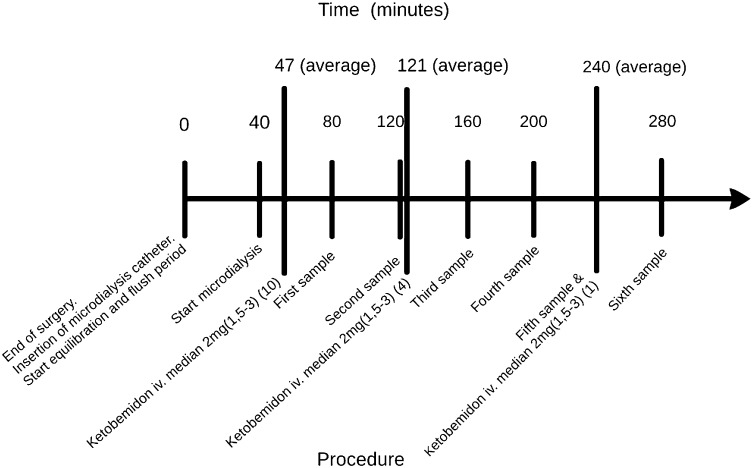
Microdialysis procedure timeline

Upon completion of the surgery 20 ml of bupivacaine with adrenaline (5 mg/ml + 5 µg/ml) was injected into the knee joint, and a microdialysis catheter with a pore size of 20 kD (CMA 60; CMA Microdialysis AB, Harvard Bioscience, Kista, Sweden) was introduced into the synovial membrane on the medial side of the knee under arthroscopic control. All catheters were perfused with a perfusion fluid (Perfusion fluid T1, CMA Microdialysis AB, Harvard Bioscience, Kista, Sweden) and the perfusion speed was 2.0 µl/min. The equilibration of the system started when a flush period of 40 min was finished. The first dialysate was collected after another 40 min. The samples were quickly frozen to minus 80 °C, and were later analysed together in the same assay for concentrations of glucose and glycerol with the CAM 600 (CMA Microdialysis AB, Harvard Bioscience, Kista, Sweden), and PGE_2_ using enzyme-linked immunosorbent assay ELISA (PGE_2_ EIA Kit—Monoclonal; Cayman Chemical company, 1180 E, Ellsworth Rd, Ann Arbor, MI 48108, USA) along with ethanol escape percentage measuring local blood flow. This was repeated five times. Microdialysis was done for a duration of 240 min (4 h) post-surgery. A total of six vials were collected from each patient (Fig. 1). Post-surgery, the patients were only allowed intake of clear fluid without sugar. No food was allowed until the last microdialysis vial was collected.

Blood flow was measured due to its regulating effect on cellular and extracellular substances through microcirculation. It was measured with the ethanol escape method (EtOH) [14, 15]. The method measures ethanol diffusion (not produced locally) from the perfusion fluid to the extracellular compartment, which increases with increasing blood flow.

### Pain measurement

Measuring pain is commonly done using the visual analogue scale (VAS) (0 = no pain, 10 = worst imaginable pain), which is a subjective grading tool. The patients were asked to assess their VAS score every 40 min as the vials were collected. The patients who estimated their pain to be severe (VAS ≥ 4) postoperatively were offered pain medication (systemic ketobemidone). The patients were then divided into two groups depending on if they requested rescue medication (RM) or not (NRM). 47 of the patients had no need of rescue medication postoperatively. Ten patients had a higher degree of pain and required rescue medication. The first dose of pain medication was given at an average time point of 47 min post-surgery: a median of 2 mg of ketobemidone (1.5–3) iv. Four of the ten patients received a second dose on request: median 2 mg (1.5–3) iv. at average time 121 min post-surgery. One of the ten patients received a third dose of 2 mg iv. at 240 min post-surgery (Fig. 1).

**Fig. 2:**
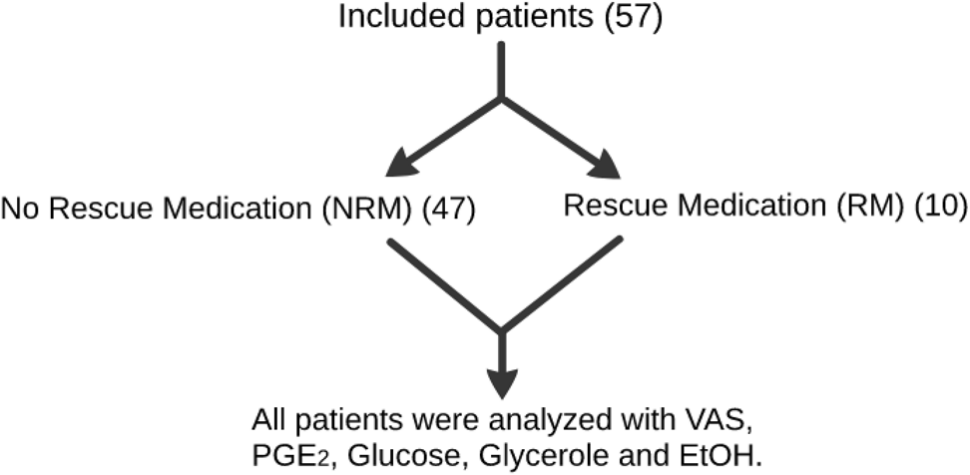
Patient flowchart

Opioids have in earlier studies shown no effect on synovial metabolism [12]. See patient demographics (Table 1) and patient flowchart (Fig. 2) for further information. The Ethics Committee of the Karolinska Institutet at the Campus Flemingsberg, Stockholm, Sweden, approved study protocols (Dnr 2017/490-31/4). All studies were conducted in adherence to the Declaration of Helsinki.

### Statistical analysis

Continuous variables are expressed as median (10–90 percentile) and nominal and ordinal variables as percentage. The continuous variables were not normally distributed hence we expressed these variables as one standard deviation increase from the mean. Significance was set at the level of *p* < 0.05. Comparisons between two groups were assessed with the non-parametric Wilcoxon test for continuous variables and Fischer exact test for nominal variables. To analyse which factors affect glucose, glycerol, and prostaglandin E_2_ variability, multivariate mixed model was used. The model includes fixed effects (patients treated with or without rescue medication) and random effects (repeated observations of glucose, glycerol, and prostaglandin E_2_). Variables showing significant univariate associations with pain were selected for a logistic regression analysis. Statistical analyses were performed using statistical software SAS version 9.4 (SAS Campus Drive, Cary, NC, USA).

## Results

**Fig. 3:**
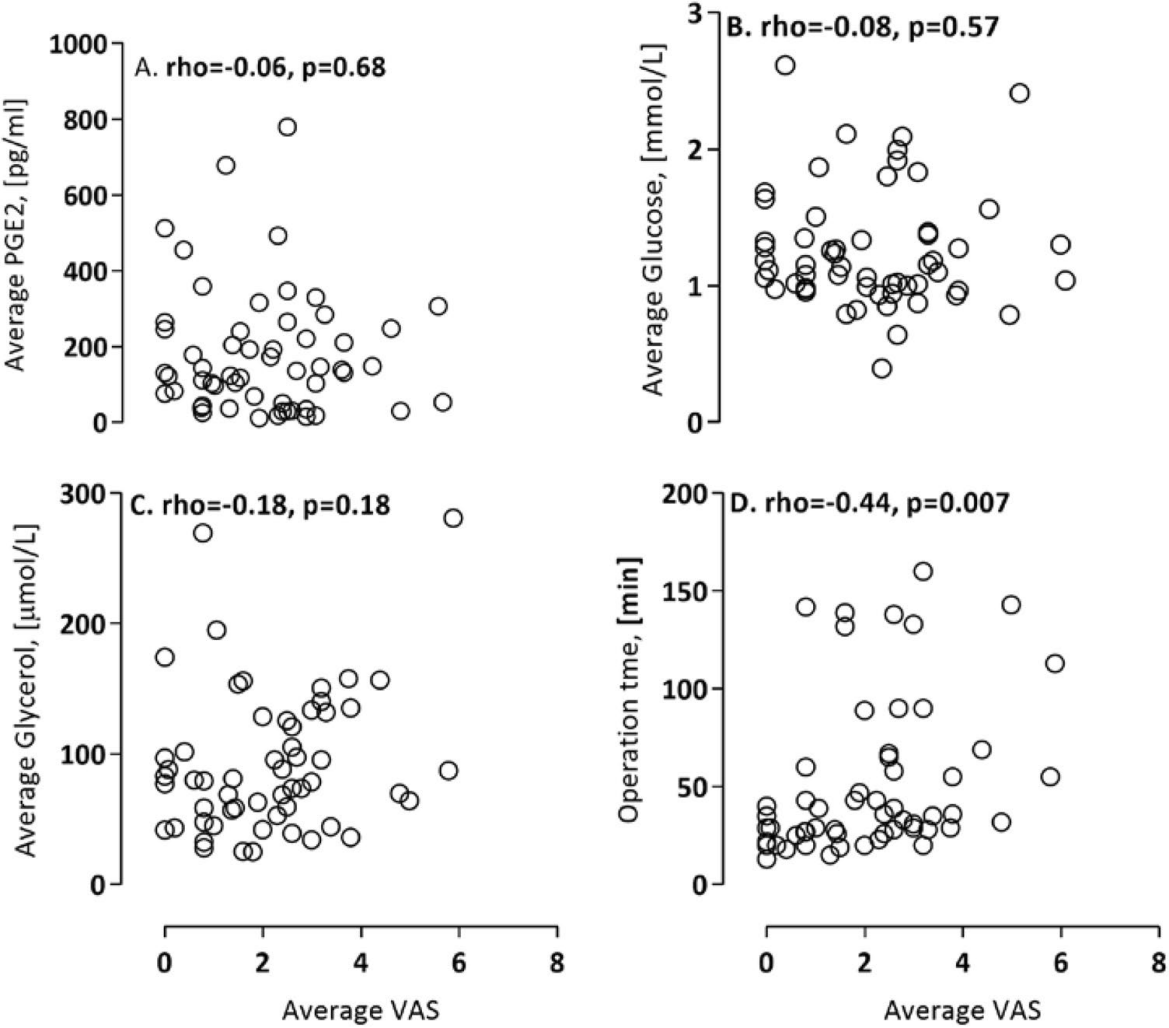
Correlation analysis between pain (VAS), duration of surgery (operation time min), PGE_2_, glucose and glycerol. There was a significant correlation between pain (VAS) and duration of surgery (*p* = 0.007), but no significant correlations between PGE_2_, glucose or glycerol and VAS

The patient characteristics are presented according to groups of patients with and without rescue medication (VAS ≥ 4), based at study start (Table 1). We found no statistical differences for ACL, tourniquet, cryo cuff and osteoarthritis between RM versus NRM patients (Table 1). Patients with rescue medication were significantly younger (*p* = 0.04), with a lower body weight (*p* = 0.02), a lower BMI (*p* = 0.04) and a higher frequency of smoking (*p* = 0.02). There was a significant correlation between pain (VAS) and duration of surgery (*p* = 0.007) (Fig. 3). There were no significant correlations between PGE_2_, glucose or glycerol and VAS (Fig. 3).

### Factors associated with pain

In a multivariate logistic regression analysis, we analysed if different factors could associate with the variation of pain (Table 2). The results showed the added predictive value of using 1-SD increase of age (*p* = 0.02), gender (*p* = 0.15), smoking (*p* = 0.01) and 1-SD increase of BMI (*p* = 0.38) (Table 2). The total multivariate logistic regression was explained by pseudo-*r*^2^ = 0.24.

**Table 2:** Significant predictors of pain in a multinomial logistic regression (*n* = 57). Pseudo *r*^2^ = 0.24; *p* = 0.003

Parameter	Estimate	Standard error	*p* values
Presence of smoking	− 1.34	0.54	0.01*
Gender (female vs male)	0.70	0.49	n.s
1-SD increase of age (years)	− 1.63	0.73	0.02*
1-SD increase of BMI (kg/m^2^)	− 0.62	0.71	n.s

Statistical significance (*) was set at the level of *p* < 0.05. Results show the added predictive value of using 1-SD increase of age (*p* = 0.02), and smoking (*p* = 0.01)

### Variability analysis

Factors that were associated with variability of VAS were evaluated using a multivariate mixed model. VAS showed a significant difference in subjective pain between the RM and NRM at 40 and 80 min (*p* = 0.0001), with the RM group experiencing a higher degree of subjective pain. The curves differed significantly (< 0.0001).

**Fig. 4:**
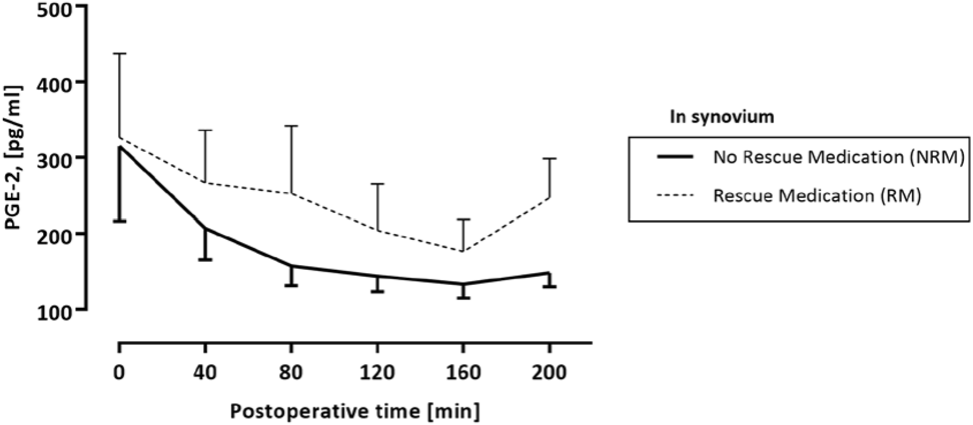
Difference in PGE_2_ between NRM group (without opioids) and RM group (with opioids) over time. Statistical significance (*) was set at the level of *p* < 0.05. The visual difference that can be noted between the curves of the RM and NRM groups failed to reach statistical significance. However, there is a statistically significant decline of PGE_2_ levels between 40 min and 120–240 min in both groups post-surgery

Although a visual difference between curves, there was no statistically significant difference between the RM and NRM groups when using multivariate mixed model for the levels of PGE_2_. However, we found a statistically significant difference between the PGE_2_ levels at 40 min and the PGE_2_ levels between 120 and 240 min (Fig. 4).

The multivariate mixed models of glucose, glycerol and EtOH showed no statistically significant differences in or between groups. Overall the synovial tissue blood flow was stable.

## Discussion

The most important finding of this study was that patients requiring rescue medication after arthroscopic knee surgery differed significantly in age, weight, BMI and smoking compared to those not requiring rescue medication. Multinomial logistic regression, showed a higher frequency of pain among young patients and smokers. These findings are in accordance with earlier studies [3, 5]. The differences were due to physiological dissimilarities, or socioeconomic factors such as social status and education level in the different age groups and among smokers/non-smokers [3–5]. To properly research these disparities, further studies are needed. In contrast with an earlier study, we found no significant difference between genders [7]. An expected finding was a significant association between duration of surgery and degree of pain. There were furthermore no statistically significant differences between the RM and NRM groups regarding type of surgery (Table 1). It could, therefore, be speculated that surgical manipulation of tissue for a longer period of time would cause a higher level of pain due to increased tissue trauma. Interestingly, the increased pain was not reflected by subsequent detectable changes in the measured inflammatory and metabolic markers. Glucose consumption, interpreted as a sign of a hypermetabolic state [9, 10], glycerol, a marker of cell membrane degradation, and therefore if increased a marker of cell death and tissue injury [16, 17], and PGE_2_, a mediator of central and peripheral pain sensitization [18]. The results also demonstrate how subjective pain measured by VAS declined with time post-surgery. VAS exhibited an initial significant difference between RM and NRM groups as was predicted and the base for this study. PGE_2_, known as a pain mediator and a marker of inflammation, showed a visual difference between the NRM and the NRM group (Fig. 4). However, this did not reach statistical significance. This result is not in accordance with earlier studies that showed an increase of PGE_2_ in patients requiring opioids [10]. The decline with time in PGE_2_ was significant in both groups post-surgery. This suggests a primary elevation associated with the initial pain and/or surgical trauma. Glucose levels showed no statistical difference between RM and NRM groups which is in contrast with other studies that demonstrate an increased consumption of glucose in the synovial membrane after minor arthroscopic surgery, but no change after ACL reconstruction [10, 13, 19]. Glycerol, the marker of cell death and cell wall degradation exhibited no difference between groups. These findings along with the finding of a higher level of pain during long, but not necessarily complex surgeries lead us to the following speculations. First, that harm done to the synovial cells is irrespective of the magnitude of the knee arthroscopic procedure itself. Second, that postoperative pain is due to extension of the knee cavity caused by the pressure of irrigation fluid, and not primarily due to the surgical trauma itself. Arthroscopy for a long period of time, with a corresponding time of distension, could, therefore, result in more pain. Limitations are that the study is based on cross-sectional data for prevalent patients undergoing knee arthroscopy. Also, there is a smaller number of patients in the RM group compared to the NRM group, which suggests a generally low level of pain caused by arthroscopic surgery.

## Conclusions

The results of the present study show a higher degree of pain in younger patients, smokers, and after long-lasting arthroscopic surgery of the knee. This should be taken into consideration during planning and management of arthroscopic surgery to create a more effective and personalized pain management.
